# Two Decades of Insights into Nontuberculous Mycobacterial Hand Infections

**DOI:** 10.1093/ofid/ofae152

**Published:** 2024-03-28

**Authors:** Hussam Tabaja, Humza Y Saleem, Karim Bakri, Aaron J Tande

**Affiliations:** Division of Public Health, Infectious Diseases and Occupational Medicine, Department of Medicine, Mayo Clinic, Rochester, Minnesota, USA; Department of General Surgery, Mayo Clinic, Jacksonville, Florida, USA; Department of Plastic Surgery, Mayo Clinic, Rochester, Minnesota, USA; Division of Public Health, Infectious Diseases and Occupational Medicine, Department of Medicine, Mayo Clinic, Rochester, Minnesota, USA

**Keywords:** hand, infection, mycobacteria, nontuberculous

## Abstract

**Background:**

The objective of our study is to describe the clinical presentation, management, and outcome of a large cohort with nontuberculous mycobacteria (NTM) hand infection.

**Methods:**

We reviewed the medical records of all adults (≥18 years) managed at the Mayo Clinic (Rochester, MN) for NTM hand infection between 1998 and 2018.

**Results:**

Our cohort included 81 patients. The median age was 61.3 (interquartile range 51.7, 69.6) years; 39.5% were immunocompromised, and 67.9% reported a triggering exposure preceding infection. Infection was deep in 64.2% and disseminated in 3.7%. Up to 16.0% received intralesional steroids because of misdiagnosis with an inflammatory process. Immunocompromised patients had deeper infection, and fewer reports of a triggering exposure. *Mycobacterium marinum*, *Mycobacterium avium* complex, and *Mycobacterium chelonae/abscessus* complex were the most common species. The median antibiotic duration was 6.1 (interquartile range 4.6, 9.9) months. Deep infection and infection with species other than *M marinum* were associated with using a greater number of antibiotics for combination therapy and an extended duration of treatment. Immunosuppression was also associated with longer courses of antibiotic therapy. Surgery was performed in 86.5% and 32.4% required multiple procedures. Ten patients, mostly with superficial infections, were treated with antibiotics alone. The 5-year cumulative rate of treatment failure was 30.3% (95% confidence interval, 20.9–44.0). Immunosuppression and intralesional steroid use were risk factors for failure.

**Conclusions:**

Treatment of NTM hand infection usually requires surgery and antibiotics, but antibiotics alone may occasionally be attempted in select cases. Immunosuppression and intralesional steroids are risk factors for treatment failure.

## Teaser

- Effective treatment for nontuberculous mycobacteria hand infection involves surgery and prolonged antimicrobial courses.
- Misdiagnosis of nontuberculous mycobacteria hand infection may lead to use of intralesional steroids.
- Intralesional steroids use and immunosuppression are risk factors for failure.
- Five-year cumulative failure rate can reach 30%.

Nontuberculous mycobacteria (NTM) are ubiquitously found across various environmental niches [1]. To date, more than 150 NTM species have been identified, some of which are now known to cause human infection [2]. Although pulmonary infection is the most common human NTM disease, almost 12% of infections involve extrapulmonary sites [3]. NTM hand infection is 1 type of extrapulmonary disease that has received increased attention over the years despite its overall low reported incidence [3–6]. This is due to the diagnostic and therapeutic challenges of NTM hand infection and its associated morbidity [5]. Furthermore, referral centers with experienced hand surgeons and infectious diseases specialists are already seeing higher volumes of cases of NTM hand infections because of an aging population and higher rates of iatrogenic immunosuppression. Although this is true, our understanding of this entity remains poor because of the scarce literature. Our hospital system provided care to a number of patients with NTM hand infection over the years. A prior report from the Mayo Clinic enterprise described a cohort of 76 patients with “non-marinum” NTM infection of the upper extremity (shoulder to fingertips) between 2011 and 2018 [7]. In this present study, we add to the previous research by including a larger group of patients with infection specifically restricted to hand or wrist, encompassing all species, and examining a longer study period. Our objective is to provide a comprehensive description of the microbiological profile, treatment strategies, and factors influencing the outcome.

## METHODS

Following institutional review board approval, a retrospective chart review was performed for all patients diagnosed with NTM infection of the hand or wrist at the Mayo Clinic (Rochester, MN) between 1998 and 2018. Criteria for diagnosis were adapted from the American Thoracic Society (ATS) guidelines for NTM disease [2]. Accordingly, patients with compatible clinical findings and 1 or more sterile site cultures for an NTM organism were included in this cohort. Additionally, in the absence of mycobacterial growth, patients with compatible clinical findings and positive histopathology for granulomatous inflammation and acid-fast bacilli were included. A patient list was acquired from the central microbiology laboratory. Patients <18 years of age and patients with NTM infections localized proximal to the wrist were excluded. In our facility, patients with NTM hand infection receive assessment and ongoing care in a unified clinic where they are attended to by both an orthopedic infectious diseases specialist and a hand surgeon. Throughout the study duration, a minimum of 11 infectious diseases physicians, 9 hand surgeons, and a small team of advanced practice providers and clinical pharmacists participated in the care of patients with NTM hand infections.

Data on patient demographics, comorbidities, pertinent exposures before infection, depth of infection, and mycobacterial species were collected and described. Also, time from symptom onset to microbiologic diagnosis was measured to highlight chronicity of symptoms before diagnosis. However, the date of first medical encounter for hand NTM was unknown for most of the patients referred from outside institutions. As such, the time from presentation to diagnosis (ie, diagnostic delay) was not measured in our study. Immunosuppression was defined as having any of the following: primary or acquired immunodeficiencies, HIV, bone marrow or solid organ transplant, and receipt of immunosuppressants. For chronic steroid use, an equivalent dose ≥15 mg prednisone daily for ≥1 month was considered immunosuppressing. Depth of infection was classified into 2 groups: (1) superficial infection, defined as infection limited to skin and soft tissue, or (2) deep infection, defined as the occurrence of 1 or more of tenosynovitis, septic arthritis, or osteomyelitis. Disseminated infection was defined as the isolation of NTM in culture from blood or sterile specimen obtained from a site distant to the infected hand or wrist.

Treatment was defined as the start of antibiotic therapy against NTM. Although surgery remains an essential part of therapy for most patients, we did not consider surgery an obligatory part of therapy because some patients with NTM hand infection may attempt medical management alone. Treatment failure was the primary outcome of interest. As such, the outcome follow-up started from the date of antibiotic start and continued until incident treatment failure or lost to follow-up. Treatment failure was defined as the persistence or recurrence of infectious symptoms requiring repeat surgery or change in antibiotic regimen ≥3 months from the original antibiotic start date. We chose a 3-month lapse from therapy before determining treatment outcome because clinical response in NTM hand infection is typically slow. In a systematic review of 241 NTM hand infections, the first sign of clinical improvement was observed after a median of 3 months [8]. Although the systematic review excluded deep infections, we believe that treatment response should become evident after 3 months for deep infections as well. In patients with treatment failure, a positive culture demonstrating the same NTM species or a positive histopathology for acid-fast bacilli was considered a confirmed microbiologic failure.

Our secondary outcome of interest was a composite outcome of amputations, tenosynovectomies, or synovectomies performed either as part of planned therapy or as response to treatment failure. Such procedures may be curative of infection but have negative cosmetic and functional effect; hence, they were measured to assess disease burden.

Frequency counts and percentages were used for categorical variables, whereas medians (interquartile ranges [IQRs]) were used for continuous variables. For antibiotic therapy duration, the median was estimated using Kaplan-Meier (KM) estimator curve for time to antibiotic stop. On the other hand, the follow-up time for treatment outcome was estimated using reverse KM estimator curve for time to censoring (ie, lost to follow-up was treated as the event). Group comparisons were made using the Pearson chi-square or Fisher exact test for categorical variables; the nonparametric Wilcoxon-Mann-Whitney test was used for continuous variables when comparing 2 independent samples or Kruskal-Wallis test for ≥3 independent samples. A Cox regression model was fitted for the risks of treatment failure. The model was adjusted for immune status, depth of infection, intralesional steroid injection, and number of antibiotics used for therapy. Based on our definition for treatment failure, only patients who completed at least 3 months of follow-up from the date of antibiotic start were included in the Cox model. *P* values < .05 were considered significant. Statistical analysis was performed using XLSTAT software (version 2022.5.1; Addinsoft, New York, USA) [9].

## RESULTS

### Demographics and Clinical Presentation:

Eighty-one patients had NTM infection of either the hand or wrist (Supplementary Figure 1). The median age at the time of diagnosis was 61.3 (IQR 51.7, 69.6) years. Fifty-three (65.4%) patients identified as male and 78 (96.3%) as White. Table 1 summarizes their baseline characteristics and triggering exposure preceding NTM hand infection; 32 (39.5%) patients were immunocompromised, 7 (8.6%) had rheumatoid arthritis, and 55 (67.9%) reported a triggering event preceding infection. For patients who did not report a trigger for NTM hand infection, this could be attributed to the patient's inability to identify or recall a trigger or to inadequate history gathering and documentation.

**Table 1.** Baseline Characteristics of Patients With NTM Hand Infection (N = 81)

Diabetes mellitus, n (%)	13 (16.0)
Cancer, n (%)	3 (3.0)
Autoimmune disease, n (%)	13 (16.0)
Rheumatoid arthritis, n	7
Systemic lupus erythematosus, n	2
Crohn disease, n	1
Other, n	3
Immunocompromised, n (%)	32 (39.5)
Solid transplant (SOT), n	8
Renal, n	4
Renal-pancreas, n	3
Heart, n	1
Immunosuppressive therapy preceding NTM infection, n (%)	26 (32.1)
Therapy for SOT, n	8
Therapy for rheumatoid arthritis, n	7
Therapy for other autoimmune diseases, n	8
Chemotherapy for cancer, n	3
HIV, n (%)	1 (1.2)
Any significant exposure before infection,^a^ n (%)	55 (67.9)
Aquatic exposure,^b^ n	19
Gardening, n	20
Any known injury, n	35
Animal bite, n	4
Water-related injuries, n	5
Garden-related injuries, n	5
Other cuts/injuries, n	22

Categorical variables are reported using frequency counts (column percentages).

Abbreviation: NTM, nontuberculous mycobacteria.

^a^Individual patients could have multiple exposures; hence, subtotals add up to >55.

^b^Included swimming in fresh or marine water, fish-related injuries, or handling a fish tank.

The median time from symptom onset to microbiologic diagnosis was 4.4 (IQR 2.0–7.8) months. At least 17 (21.0%) patients received therapy for misdiagnosis of “inflammatory arthritis,” 12 of which received intralesional steroids, 4 received systemic immunosuppressants, and 1 received both. Infection involved 1 or more digits in 53 (65.4%) patients, the hand in 41 (50.6%) patients (16 with palmar and 25 with dorsal infection), and the wrist in 25 (30.9%) patients. At least 34 (42.0%) patients had infection extending over 2 or more anatomic sites. In 29 (35.8%) patients, the infection was superficial. The remaining 52 (64.2%) patients had deeper infection including tenosynovitis in 41 (50.6%), septic arthritis in 10 (12.3%), and osteomyelitis in 9 (11.1%), with some patients overlapping. There were only 3 (3.7%) patients with disseminated disease. Only 1 (1.2%) patient had hardware-associated infection involving proximal interphalangeal joint arthrodesis for rheumatoid arthritis.

Supplementary Table 1 compares the clinical presentation between immunocompromised and immunocompetent patients. Immunocompetent patients were more likely to report a triggering event preceding NTM infection (*P* = .002) and have a higher rate of superficial infection compared with immunocompromised patients (*P* = .010).

### Microbiology

Eighty patients were diagnosed based on at least 1 positive mycobacterial culture from hand or wrist specimen, whereas 1 patient was diagnosed based on 2 specimens showing granulomatous inflammation and positive acid-fast bacilli stain. For the 80 patients with culture-proven disease, a total of 84 mycobacterial isolates were recovered: 79 patients had 1 isolate each, whereas 1 patient had 5 isolates. In 1 patient, our central laboratory was unable to identify the mycobacterial species. On the other hand, species identification was available for the remaining 83 isolates recovered from 79 patients. Twenty-four (30.4%) of these patients had rapidly-growing mycobacteria (RGM) and 56 (70.9%) had slowly-growing mycobacteria (SGM) (ie, 1 patient had both). There was no significant difference in delay from symptom onset to microbiologic diagnosis between patients with RGM and SGM infections (3.7 [IQR 2.3, 6.9] months versus 5.1 [IQR 2.3, 8.0] months; *P* = .273).

**Figure 1.**
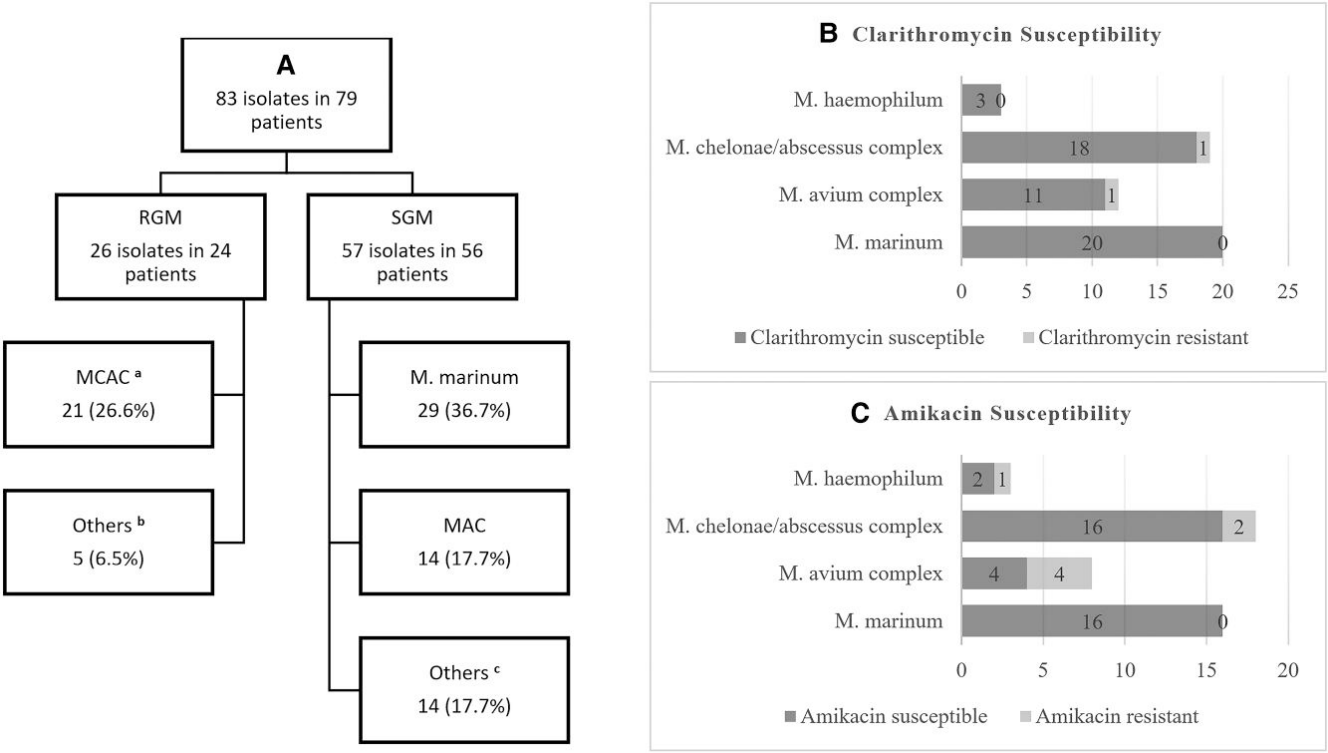
*A*, Mycobacterial species distribution for 79 patients with species identification. Bracket values are percentages of 79 patients. Because 1 patient had multiple isolates, percentages do not add up to 100.0%. MCAC, *M chelonae/abscessus* complex; MAC, *M avium* complex. ^a^Includes *M abscessus* isolate (n = 1), *M chelonae* isolates (n = 8), and *M chelonae/abscessus* complex not further identified (n = 12). ^b^Other species included 3 *M fortuitum* complex, 1 *M iranicum*, and 1 *M mucogenicum*. ^c^Other species included 4 *M haemophilum*; 2 each of *M kansasii*, *M szulgai*, *M arupense*, and *M gordonae*; and 1 each of *M xenopi* and *M nebraskense*. *B* and *C,* The clarithromycin and amikacin susceptibilities for common isolates. Susceptibilities were not documented for all isolates; hence, the numbers do not add up to the total number of recovered isolates. For susceptibility reporting, isolates identified as *M abscessus*, *M chelonae*, or *M chelonae/abscessus* complex are all combined under *M chelonae/abscessus* complex.

Figure 1 describes the species distribution for the 79 patients in whom species identification was available. *Mycobacterium marinum* was the most identified species, isolated in 29 (36.7%) patients. Twenty-one (26.6%) patients had *Mycobacterium chelonae/abscessus* complex (MCAC), further identified as *Mycobacterium abscessus* in 1 patient, and *M chelonae* in 8. Whether the remaining 12 MCAC isolates represent *M chelonae*, *M abscessus*, or other species within the complex is undetermined. *Mycobacterium avium* complex (MAC) was identified in 14 (17.7%) patients. Notably, 16 (55.2%) of 29 patients with *M marinum* reported exposure to fresh or marine water organisms. Of the 3 patients with disseminated infection, 2 had *Mycobacterium haemophilum* and 1 had MCAC.

Supplementary Table 2 stratifies species distribution according to depth of infection and immune status. There was no difference in distribution of RGM and SGM based on immune status (*P* = .528) or depth of infection (*P* = .635). However, at the species level, immunocompromised patients had higher rates of infection with MAC compared with immunocompetent patients (*P* < .001). In contrast, immunocompetent patients had higher rates of *M marinum* infections (*P* < .001). Both groups had comparable rates of MCAC (*P* = .099). Patients with deep infections had higher rates of MAC infection and lower rates of *M marinum* compared with those with superficial infections; however, this was not statistically significant (*P* = .263). There was also no statistically significant difference in distribution of other species between both groups.

Finally, 20 (24.7%) of the 81 patients had isolation of other bacteria (n = 18) or fungi (n = 6); however, these were considered of clinical significance and treated in only 10 (12.3%) patients.

### Therapeutic Approach

Overall, 74 patients completed at least 3 months of follow-up from antibiotic start date. Hereafter, our treatment and outcome analysis will be restricted to these 74 patients. Sixty-one (82.4%) patients were diagnosed and started on treatment at our institution, whereas the remaining 13 (17.6%) were referred to our hospital for second opinions after they were diagnosed in an outside facility.

Sixty-four (86.5%) of the 74 patients underwent debridement and nodule or mass excision for infection within 3 months from antibiotic initiation. The median time from symptom onset to the first surgical procedure was 4.1 (IQR 2.3, 7.6) months. Twenty-four (32.4%) patients required multiple surgeries, with a median of 2.0 (IQR 2.0, 3.0) and a range of 2–11 procedures. On the other hand, 10 (12.3%) patients were treated without surgery (8 superficial infections, 1 osteomyelitis, and 1 tenosynovitis). Table 2 divides the treated patients into surgical and nonsurgical groups. Patients requiring at least 1 surgical intervention within 3 months from antibiotic initiation had a higher rate of deep infection compared with those treated with antibiotics alone (*P* = .007). Otherwise, there was no significant difference in immune status or type of NTM species between the surgical and nonsurgical groups.

**Table 2.** Characteristics of Therapy Subgroups for NTM Hand Infection (N = 74)

	Maximum Number of Antibiotics Used				Number of Surgical Interventions			
	Single-drug	Multidrug			Nonsurgical	Surgical		
	1 (n = 15)	2 (n = 17)	≥3 (n = 42)	*P* Value	0 (n = 10)	1 (n = 40)	≥2 (n = 24)	*P* Value
Deep infection	5 (33.3%)	8 (47.1%)	35 (83.3%)	.001	2 (20.0%)	29 (72.5%)	17 (70.8%)	.007
Immunocompromised	5 (33.3%)	4 (23.5%)	20 (47.6%)	.200	4 (40.0%)	16 (40.0%)	9 (37.5%)	1.000
Mycobacterial growth pattern, SGM	11 (73.3%)	8 (47.1%)	31 (73.8%)	.144	6 (60.0%)	29 (72.5%)	15 (62.5%)	.720
Mycobacterial species	-	-	-	.030	-	-	-	.640
* M marinum*	10 (66.7%)	6 (35.3%)	10 (23.8%)	-	5 (50.0%)	14 (35.0%)	7 (29.2%)	-
MAC	0	2 (11.8%)	10 (23.8%)	-	0	9 (22.5%)	3 (12.5%)	-
MCAC	4 (26.7%)	6 (35.3%)	9 (21.4%)	-	3 (30.0%)	9 (22.5%)	7 (29.2%)	-
Others	1 (6.7%)	3 (17.6%)	13 (31.0%)	-	2 (20.0%)	8 (20.0%)	7 (29.2%)	-
Surgery				.003	-	-	-	-
1 procedure	7 (46.7%)	12 (70.6%)	21 (50.0%)	-	-	-	-	-
≥2	2 (13.3%)	3 (17.6%)	19 (45.2%)	-	-	-	-	-

Categorical variables are reported using frequency counts (column percentages) and compared using Pearson chi-square or Fisher exact test, as appropriate. Continuous variables are reported using medians (interquartile range) and compared using the nonparametric Kruskal-Wallis test.

Abbreviations: MAC, Mycobacterium avium complex; MCAC, Mycobacterium chelonae/abscessus complex; NTM, nontuberculous mycobacteria; SGM, slowly growing mycobacteria.

Table 2 also categorizes treated patients into single-drug and multidrug groups based on maximum number of antibiotics prescribed during treatment. Fifteen (20.3%) patients completed the entire therapy course with a single antibiotic, whereas a multidrug regimen consisting of 2 antibiotics was used in 17 (23.0%) patients, and 3 or more antibiotics in 42 (56.8%) patients. In the multidrug group, only 8 patients were later transitioned to a single antibiotic to finish therapy. Compared with the single-drug group, the multidrug group had a higher rate of deep infection (*P* = .001). They also had a higher rate of infection with species other than *M marinum* (*P* = .030). Otherwise, there was no significant difference in immune status. Importantly, patients treated with multiple antibiotics required a higher number of surgical interventions compared with those treated with 1 antibiotic (*P* = .003), suggesting more severe infection in the former group.

The median total duration of antibiotic therapy for the entire cohort was 6.1 (IQR 4.6, 9.9) months. Supplementary Figure 2 shows KM curves for antibiotic duration for subgroups divided based on infection depth, immune status, number of antibiotics prescribed, species type, and surgical management. Longer courses of antibiotics were prescribed for immunocompromised compared with immunocompetent patients (*P* = .003) and for patients with deep infection compared with those with superficial infection (*P* = .026). Patients treated with multiple antibiotics were also prescribed longer courses compared with those treated with a single antibiotic (*P* = .002). The difference in length of treatment remained statistically significant when comparing patients treated with 3 or more antibiotics to those treated with fewer than 3 antibiotics (*P* = .002). Furthermore, patients infected with non-marinum species received longer therapy than those with *M marinum* (*P* = .023). As previously outlined, non-marinum species were more commonly detected in immunocompromised patients, which may partly explain the longer therapy prescribed in these patients.

Supplementary Table 3 lists the antibiotics used to treat the 3 most common organisms identified in our study. Of the 26 patients treated for *M marinum*, 10 received a single-drug program that commonly included a macrolide, or trimethoprim-sulfamethoxazole, or a tetracycline drug. Ten other patients were treated with 3 or more drugs, of whom 7 received a regimen of macrolide plus ethambutol and rifamycin and 2 received a macrolide plus rifamycin and doxycycline. Six patients received a 2-drug program, with most combinations including a macrolide, with the addition of either a rifamycin, trimethoprim-sulfamethoxazole, doxycycline, or a fluoroquinolone. All 12 patients in the treated MAC group received a multidrug program. Ten received 3 or more antibiotics that included a macrolide plus ethambutol and rifamycin, with the addition of a fluoroquinolone and/or amikacin in 2 patients. Two other patients received 2-drug therapy with a macrolide and ethambutol. Finally, of the 19 patients treated for MCAC, 4 received a single-drug program consisting of a macrolide. The combination therapy used for MCAC was quite variable. All 9 patients receiving 3 or more antibiotics were placed on a macrolide at some point of their treatment. The most common companion medications included tigecycline, fluoroquinolones, imipenem, and aminoglycosides. Six other patients received a 2-drug program, all treated with a macrolide in combination with either a fluoroquinolone, linezolid, trimethoprim sulfamethoxazole, or tigecycline.

Finally, 25 (33.8%) patients encountered intolerances or toxicities necessitating a switch in antibiotics, underscoring the challenging nature of NTM antimicrobial therapy.

### Treatment Outcome

The median follow-up time from start of antibiotics was 6.0 (IQR 1.3, 9.5) years. During follow-up, 20 patients failed treatment. The 5-year cumulative treatment failure rate was 30.3% (95% confidence interval, 20.9–44.0), respectively (KM curves in Supplementary Figure 3). Two of the 10 patients treated medically and 18 of those treated with surgery failed therapy; the difference was not statistically significant (*P* = .614) (KM curve not shown). Microbiologic failure with positive cultures or histopathology at time of clinical failure was confirmed in 9 (12.2%) patients only.

Table 3 describes some of the risk factors for treatment failure. Based on an unadjusted Cox proportional hazards model, immunocompromised patients, patients with deep infection, and patients who received intralesional steroid injection were at higher risk for treatment failure. However, when adjusting for immune status, depth of infection, steroid injection, and treatment with 3 or more antibiotics, only immunosuppression and intralesional steroid injection remained associated with a higher risk of failure (Supplementary Table 4 shows the full adjusted model).

**Table 3.** Risk Factors for Treatment Failure in Hand NTM Infection (N = 74)

	Unadjusted Cox Regression Model			Adjusted Cox Regression Model	
	No Failure (n = 54)	Treatment Failure (n = 20)	*P* Value	Hazard Ratio (95% confidence interval)	*P* Value
Age at diagnosis, y	59.8 (48.7, 69.6)	62.9 (57.0, 68.2)	.593	-	…
Diabetes mellitus	8 (14.8)	4 (20.0)	.469	-	…
Immunocompromised^a^	16 (29.6)	13 (65.0)	.018	2.78 (1.04–7.40)	.041
Deep infection^a^	32 (59.3)	17 (85.0)	.028	-	…
Polymicrobial infection^b^	8 (14.8)	2 (10.0)	.678	-	…
Mycobacterial growth pattern, SGM	36 (66.7)	14 (70.0)	.998	…	…
Mycobacterial species					
* M marinum*	20 (37.0)	6 (30.0)	Ref	-	…
* *MAC	6 (11.1)	6 (30.0)	.310	-	…
* *MCAC	13 (24.1)	6 (30.0)	.719	-	…
* *Others	15 (27.8)	2 (10.0)	.292	-	…
Intralesional steroid injection^a,c^	5 (9.3)	7 (35.0)	.007	3.56 (1.35–9.40)	.010
≥3 antibiotics^a^	27 (50.0)	15 (75.0)	.079	-	…
Surgical management	46 (85.2)	18 (90.0)	.616	-	…
Antibiotics changed for intolerance	16 (29.6)	9 (45.0)	.313	-	…

Categorical variables are reported using frequency counts (column percentages). Continuous variables are reported using medians (interquartile range). A Cox proportional hazards model was fitted for risk factor analysis.

Abbreviations: MAC, Mycobacterium avium complex; MCAC, Mycobacterium chelonae/abscessus complex; NTM, nontuberculous mycobacteria; SGM, slowly growing mycobacteria.

^a^The adjusted model controlled for immune status, depth of infection, intralesional steroid injection, and ≥3 antibiotics.

^b^Including only those considered clinically significant.

^c^Steroid injection administered for a misdiagnosis of inflammatory arthritis before making the diagnosis of NTM infection.

Additionally, 33 (44.6%) patients met our secondary outcome. Specifically, 13 (27.6%) patients required amputations, 20 (27.0%) required tenosynovectomies, and 3 (4.1%) required synovectomies. Immunocompromised patients were more likely to undergo such procedures compared with immunocompetent patients (69.0% vs 28.9%; *P* = .001).

## DISCUSSION

Our study is important because it is the largest series examining NTM infection localized to the hand and wrist in the United States [4, 6, 8, 10–13]. We did not limit our examination based on depth of infection or species type. Our cohort largely consisted of elderly patients presenting with subacute-to-chronic symptoms. Infection was commonly deep but rarely disseminated. Immunosuppression was associated with more severe disease. The most commonly identified species were *M marinum*, MAC, and MCAC. Antibiotic therapy varied considerably according to immune status, depth of infection, and species type. Surgery remained a vital part of management for most patients, but a small subgroup of patients had favorable response with medical therapy alone. Finally, immunosuppression and intralesional steroid injection were risk factors for treatment failure.

Several investigators have previously reported on NTM hand infection with heterogeneity in study design. For example, some reports included all cutaneous NTM infections or infections proximal to the hand [4, 13], some excluded septic arthritis and osteomyelitis [8], and some were restricted to specific species [6, 12]. These differences should be considered when comparing our findings with prior reports. Overall, the age distribution, and the subacute-to-chronic nature of infection seen in our cohort match the current literature [6, 8, 11–13]. Patients may wait for months from symptom onset before presenting for care, and this has been reported for both SGM and RGM species across a wide range of cutaneous NTM infections [6, 12, 14]. Although we could not measure the diagnostic delay from patient's first medical encounter in our cohort, the reported delay in diagnosis ranged from 2 to 4 months in prior series, which again reflects the indolent nature of such infections [12–14]. When patients seek medical attention, they often present to the emergency department, urgent care, or primary care/general practitioner clinics, settings not equipped to diagnose NTM hand infections [12, 13]. Therefore, many patients are misdiagnosed early during their disease. In a large systematic review of 241 patients, an incorrect diagnosis with delayed treatment occurred in 53% of patients. Given the complexity of such infection, misdiagnosis will likely continue to be an issue with significant impact on outcome.

Certain patterns of clinical presentations observed in our study resemble past observations. Up to 40% of our patients were immunosuppressed. The prevalence of immunosuppression ranged from 0% to 45% in the literature, indicating that our rate was higher than some prior reports [6, 8, 10]. In our cohort, immunocompromised patients had a higher rate of deep infection compared with immunocompetent patients. Moreover, a smaller portion of the immunocompromised patients reported a triggering event preceding NTM infection compared with the immunocompetent group. Nevertheless, these data may be influenced by inadequate history gathering and documentation or the patient's inability to recall a trigger. Healthcare providers should carefully inquire about significant triggers linked to NTM infection when evaluating patients with hand lesions and/or inflammation. Regardless of the immune status, disseminated disease was rare. These distinctive presentations were similarly documented by other investigators [4, 8, 10, 12, 13].

All prior reports agreed that *M marinum*, MAC, and MCAC are the most frequently involved organisms in NTM hand infection [4, 8, 10, 12, 13]. Several other species can be involved but typically account for a handful of cases [8]. The microbiologic pattern in our study differed based on immune status. Immunocompetent patients in our study had a wide distribution of NTM species, but *M marinum* and MCAC were most frequent. *M marinum* infection commonly occurred after aquatic exposure. Indeed, prior studies restricted to *M marinum* hand infection echoed our high rate of immunocompetency and aquatic exposure in such patients [8, 12]. On the other hand, MAC replaced *M marinum* as the most common species in our immunocompromised patients; MCAC remained the second most common.

Antibiotic therapy for NTM hand infection is necessary for cure unless the entire infection is completely removed during amputation. Courses are typically prolonged and consist of multiple drugs. This was clearly demonstrated in our study where the median total duration of antibiotics was 6 months and where 80% of patients received multiple drugs. This is also consistent with experiences from other centers [8, 12, 13]. However, therapy can vary considerably. Deep infection and infection with species other than *M marinum* were associated with using a greater number of antibiotics for combination therapy and an extended duration of treatment. Immunosuppression was also associated with longer courses of antibiotic therapy. Similar to antibiotics, surgery for source control remains a key component of the management of NTM hand infection. Up to 87% of our patients underwent surgery. Furthermore, 32% required multiple procedures. Our surgical rates are similar to what was previously published [8]. Nonetheless, medical management alone may be attempted in patients presenting with superficial infection and can lead to favorable response, as seen in 14% of our patients.

**Figure 2.**
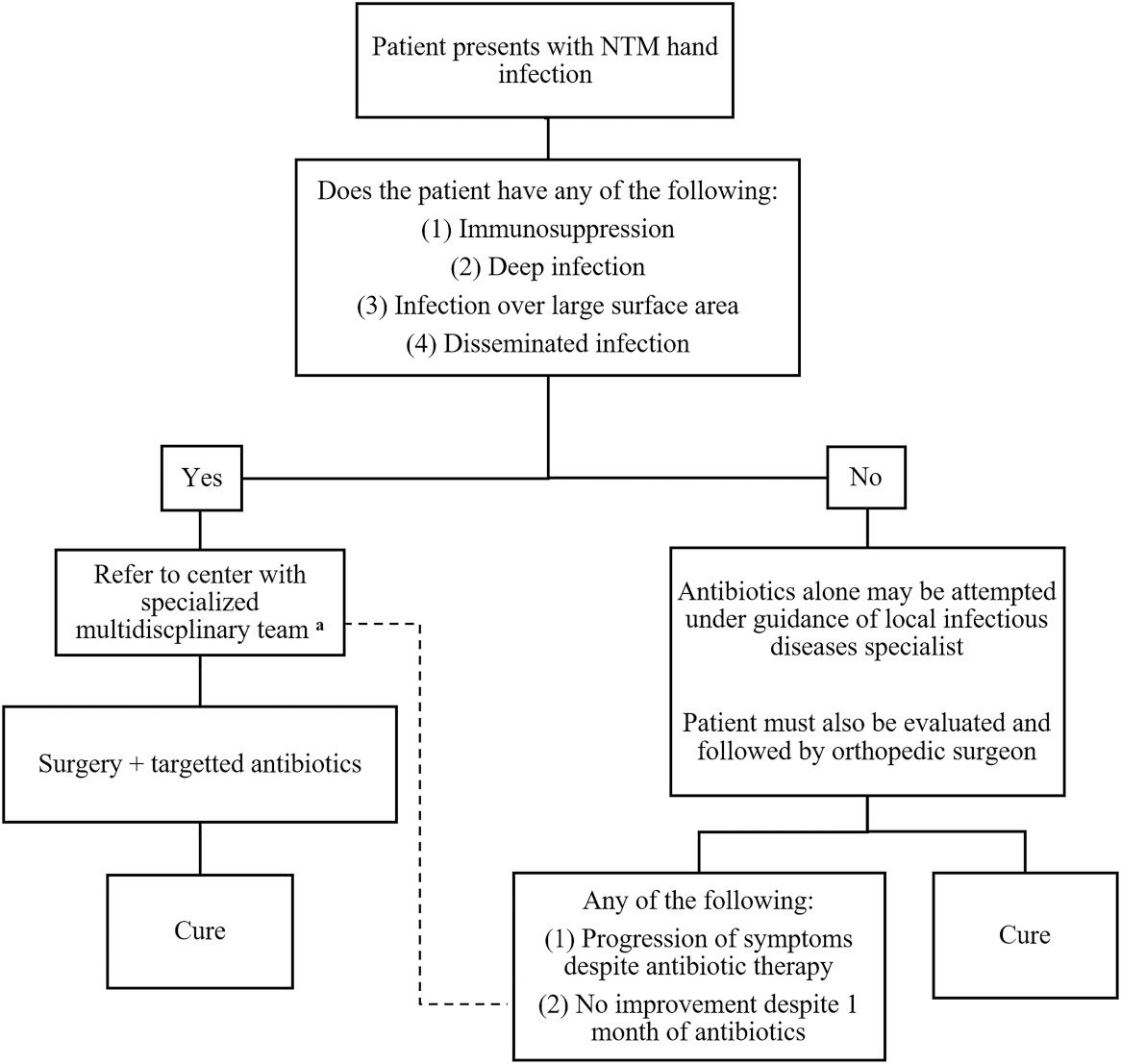
Proposed algorithm to help guide the decision for referral to a specialized center for surgery. ^a^Multidisciplinary team consisting of a hand surgeon, an infectious diseases specialist with nontuberculous mycobacteria experience, an infectious diseases pharmacist, and an outpatient antimicrobial therapy team.

The treatment of NTM hand infection is very complex and tasking. Hand surgery is a technically challenging procedure that may compromise hand function. Repeat procedures increase the risk for extensive scarring and superimposed infections; hence, risks and benefits should be carefully weighed. Antibiotics for NTM are associated with intolerances and toxicities as seen in 33.8% of our patients. Therefore, optimal therapy for NTM hand infection requires a network of specialized hand surgeons, infectious diseases specialists with NTM background, infectious diseases pharmacists, and an outpatient parenteral antimicrobial therapy program. Based on our clinical experience, we proposed an algorithm for patients with NTM hand infection to help guide decision for referral to a specialized center for surgery (Figure 2).

The 5-year cumulative failure rate reached 30% for all treated patients in our study. Despite variations in follow-up time and failure definition, reported failure rates from prior studies were very similar to ours [8, 13]. Although most of our patients achieved cure, 45% required surgeries that could have a negative cosmetic or functional effect. Our adjusted model showed higher risk of failure with immunosuppression. Cell-mediated immunity is a critical component of a host's defense against mycobacteria; hence, it is plausible that depletion of cell-mediated immunity could lead to poorly controlled NTM infection [15]. Nonetheless, this association is debated in current literature with mixed conclusions [10, 13]. Therefore, our study provides further information in favor of immunosuppression as a predictor of poor outcome. Additionally, the administration of intralesional steroids also led to worse outcomes in our cohort as well as prior cohorts [12]. In our study, 16% of patients received intralesional steroids after symptom onset and 6% received systemic immunosuppressants because of misdiagnosis as inflammatory arthritis. Intralesional steroids were used in up to 23% of patients from other studies [8]. This highlights the negative impact of misdiagnosis because it predisposes patients to therapies that may negatively impact outcome. Therefore, physicians should refrain from administering local or systemic immunosuppressants to patients presenting with hand lesions until infection is confidently ruled out.

Limitations to our study include its retrospective nature; hence, our data rely on the accuracy of documentation. Furthermore, the presented data come from a large tertiary care center specialized in treating NTM infections, which makes it prone to referral bias.

## CONCLUSIONS

Physicians across the world will continue to see an increase in NTM infections including of the hand and wrist. NTM hand infection is an entity that presents a diagnostic and therapeutic challenge, particularly in the immunocompromised host. Although cure is achievable in most patients, hand function is commonly compromised as a result of the infection and management strategies. For optimized outcome, management should be overseen by a specialized multidisciplinary team.

## Supplementary Material

ofae152_Supplementary_Data

## Supplementary Data

Supplementary materials are available at *Open Forum Infectious Diseases* online. Consisting of data provided by the authors to benefit the reader, the posted materials are not copyedited and are the sole responsibility of the authors, so questions or comments should be addressed to the corresponding author.

## Notes

***Data availability.*** Data not publicly available.

***Patient consent statement.*** Our study does not include factors necessitating patient consent.

***Financial support.*** None.
