# Supplementary material for: Two Decades of Insights into Nontuberculous Mycobacterial Hand Infections
Source: Open Forum Infect Dis. 2024 Mar 28;11(4):ofae152. doi: 10.1093/ofid/ofae152 (PMC11034953; doi:10.1093/ofid/ofae152)
Supplement: ofae152_Supplementary_Data [file ofae152_supplementary_data.docx]

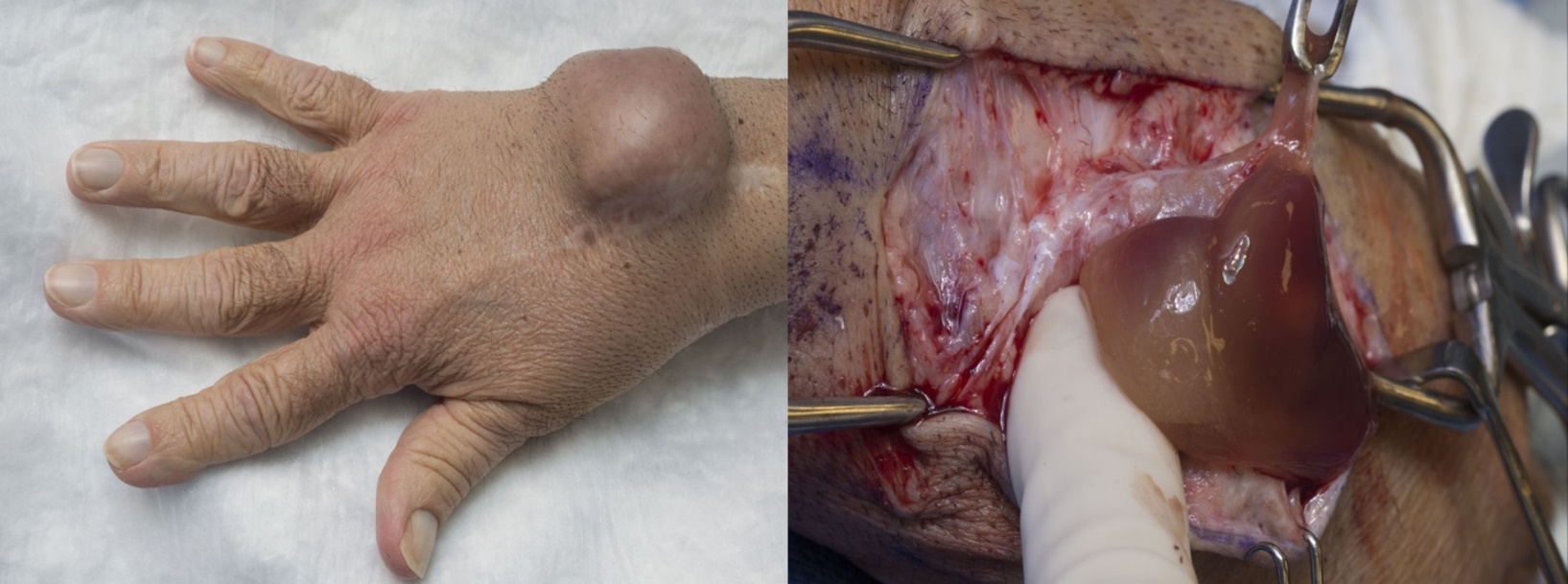


Supplementary Figure 1 - *Mycobacterium avium* complex infection of the wrist in an immunocompromised individual.

| Supplementary Table 1. Clinical Presentation Based on Immune Status (N=81). | | | |
| --- | --- | --- | --- |
|  | **Immunocompetent (N=49)** | **Immunocompromised (N=32)** | **p-value** |
| Age at time of diagnosis, years | 61.4 (50.3, 72.4) | 60.2 (52.0, 67.8) | 0.799 |
| Any significant exposure prior to infection, n (%) | 39 (79.6%) | 15 (46.9%) | **0.002** |
| Any injury, n (%) | 29 (59.2%) | 6 (18.8%) | **<0.001** |
| Exposure to fresh or marine water organisms, n (%) | 15 (30.6%) | 4 (12.5%) | 0.060 |
| Gardening, n (%) | 12 (24.5%) | 8 (25.0%) | 0.958 |
| Deep infection, n (%) | 26 (53.1%) | 26 (81.3%) | **0.010** |
| Intralesional steroid injection, n (%) | 9 (18.4%) | 4 (12.5%) | 0.482 |
| Polymicrobial growth, n (%) | 12 (24.5%) | 8 (25.0%) | 0.958 |

Categorical variables are reported using frequency counts (column percentages) and compared using Pearson’s χ2 or Fisher exact test, as appropriate. Continuous variables are reported using medians (interquartile range) and compared using the Wilcoxon-Mann-Whitney test.

| Supplementary Table 2. Mycobacterial Species Distribution (N=79). | | | | | | | |
| --- | --- | --- | --- | --- | --- | --- | --- |
| Mycobacterial Species | Superficial infection (n=28)**^a^** | Deep infection (n=51) | P-value | Immunocompromised (n=31) | Immunocompetent (n=48) **^a^** | P-value | Overall  (n=79) |
| Growth pattern |  |  | 0.635 |  |  | 0.528 |  |
| RGM, n (%) | 9 (32.1) | 15 (29.4) |  | 11 (35.5) | 13 (27.1) |  | 24 (30.4) |
| SGM, n (%) | 20 (71.4) | 36 (70.6) |  | 20 (64.5) | 36 (75.0) |  | 56 (70.9) |
| Species type |  |  | 0.263 |  |  | **<0.001** |  |
| *M. abscessus*, n (%) | - | 1 (2.0) |  | - | 1 (2.1) |  | 1 (1.3) |
| *M. mucogenicum*, n (%) | 1 (3.6) | - |  | - | 1 (2.1) |  | 1 (1.3) |
| *M. iranicum*, n (%) | - | 1 (2.0) |  | - | 1 (2.1) |  | 1 (1.3) |
| *M. nebraskense*, n (%) | 1 (3.6) | - |  | - | 1 (2.1) |  | 1 (1.3) |
| *M. xenopi*, n (%) | - | 1 (2.0) |  | - | 1 (2.1) |  | 1 (1.3) |
| *M. gordonae*, n (%) | 2 (7.1) | - |  | - | 2 (4.2) |  | 2 (2.5) |
| *M. arupense*, n (%) | - | 2 (3.9) |  | 1 (3.2) | 1 (2.1) |  | 2 (2.5) |
| *M. kansasii*, n (%) | - | 2 (3.9) |  | - | 2 (4.2) |  | 2 (2.5) |
| *M. szulgai*, n (%) | 1 (3.6) | 1 (2.0) |  | - | 2 (4.2) |  | 2 (2.5) |
| *M. fortuitum* complex, n (%) | 2 (7.1) | 1 (2.0) |  | - | 3 (6.3) |  | 3 (3.8) |
| *M. haemophilum*, n (%) | 1 (3.6) | 3 (5.9) |  | 4 (12.9) | - |  | 4 (5.1) |
| *M. chelonae*, n (%) | 3 (10.7) | 5 (9.8) |  | 7 (22.6) | 1 (2.1) |  | 8 (10.1) |
| *M. chelonae/abscessus* complex, n (%) | 5 (17.9) | 7 (13.7) |  | 4 (12.9) | 8 (16.7) |  | 12 (15.2) |
| *M. avium* complex, n (%) | 2 (7.1) | 12 (23.5) |  | 10 (32.3) | 4 (8.3) |  | 14 (17.7) |
| *M. marinum*, n (%) | 14 (50.0) | 15 (29.4) |  | 5 (16.1) | 24 (50.0) |  | 29 (36.7) |

Bracket values are column percentages. The species was not identified in 2/81 (2.5%) patients. **^a^** One patient in this category had culture growth of fiver different mycobacterial species.

**Supplementary Figure 2. (A)** Kaplan-Meier (KM) curve comparing total antibiotic duration based on the depth of infection. The total duration was longer for deep infections (Log-rank P=.026). **(B)** KM curve comparing total antibiotic duration based on immune status. The total duration was longer for immunocompromised patients (Log-rank P=.003). **(C)** KM curve comparing total antibiotic duration based on Antibiotic group. The total duration was longer for the multi-drug group (Log-rank P=.002). **(D)** KM curve comparing total antibiotic duration based on the number of antibiotics prescribed. The duration was longer for patients receiving ≥3 antibiotics than for patients receiving <3 antibiotics (Log-rank P=.002). **(E)** KM curves comparing total antibiotic duration based on species. The duration was longer for non-marinum species compared to *M. marinum* (Long-rank P=.023). **(F)** KM curve comparing total antibiotic duration based on the surgical group. No difference in duration between patients treated medically and patients requiring surgery (Log-rank P=.673).

| Supplementary Table 3. Antibiotics Used for NTM Hand Infection in Study Cohort. | | | | | | | | | |
| --- | --- | --- | --- | --- | --- | --- | --- | --- | --- |
| NTM species |  | **Single-drug** | | | | **Multi-drug** | | |  |
|  | **N** | | **Antibiotics used for monotherapy** | **Length of therapy**  **(months)** |  | **N** | **Antibiotics used for combination therapy** | **Length of therapy**  **(months)** |  |
| *M. marinum* | 10 | | Trimethoprime-sulfamethoxazole  Macrolide (CLR)  Tetracycline (MIN or DOX)  Fluoroquinolone (MXF) | 4.7 (4.6, 6.0) |  | 16 | Macrolide (CLR or AZM)  Rifamycin (RIF or RFB)  Ethambutol  Tetracycline (MIN or DOX)  Trimethoprime-sulfamethoxazole  Fluoroquinolone (MXF or LVX) | 6.0 (4.0, 9.8) |  |
| *M. avium* complex | 0 | | - | - |  | 12 | Macrolide (CLR or AZM)  Rifamycin (RIF or RFB)  Ethambutol  Amikacin  Fluoroquinolone (MXF or LVX) | 12.0 (6.0, 14.4) |  |
| *M. chelonae/abscessus* complex | 4 | | Macrolide (CLR or AZM) | 5.3 (4.1, 6.5) |  | 15 | Macrolide (CLR or AZM)  Tigecycline  Linezolid  Beta-lactam (IPM or CTX or FOX)  Fluoroquinolone (MXF or LVX or CIP)  Tetracycline (MIN or DOX) | 6.9 (4.1, 9.0) |  |

Categorical variables are reported using frequency counts (column percentages). Continuous variables are reported using medians (interquartile range). Most common macrolide used for *M. marinum* treatment was clarithromycin. Most common rifamycin used for *M. marinum* treatment was rifampin.

**Abbreviations**: **AZM**: azithromycin; **CIP**: ciprofloxacin; **CLR**: clarithromycin; **CTX**: cefotaxime; **DOX**: doxycycline; **FOX**: cefoxitin; **IPM**: imipenem; **LVX**: levofloxacin; **MIN**: minocycline; **MXF**: moxifloxacin; **RFB**: rifabutin; **RIF**: rifampin.

**Supplementary Figure 3. (A)** Reverse Kaplan-Meier (KM) curve shows the follow-up time from the start of antibiotic treatment while censoring for treatment failure. **(B)** KM curve shows the cumulative rate of treatment failure after the start of antibiotic treatment while censoring for loss to follow-up.

| **Supplementary Table 4**. Adjusted Cox Proportional Hazards Model. | | | | | | | |
| --- | --- | --- | --- | --- | --- | --- | --- |
| Variable | Value | Standard error | Wald Chi-Square | Pr > Chi² | Hazard ratio | Hazard ratio Lower bound (95%) | Hazard ratio Upper bound (95%) |
| Intralesional steroid injection | 1.270 | 0.495 | 6.568 | 0.010 | 3.560 | 1.348 | 9.401 |
| Immunocompromised | 1.021 | 0.500 | 4.163 | 0.041 | 2.775 | 1.041 | 7.395 |
| Superficial infection | -0.816 | 0.717 | 1.297 | 0.255 | 0.442 | 0.108 | 1.801 |
| ≥3 antibiotics | 0.156 | 0.595 | 0.069 | 0.793 | 1.169 | 0.364 | 3.754 |

| Goodness of fit statistics. | | |
| --- | --- | --- |
| Statistic | Independent | Full |
| Observations | 20.000 | 20.000 |
| Degree of freedom (DF) | 0.000 | 4.000 |
| -2 Log(Likelihood) | 161.739 | 145.752 |
| Akaike’s information criterion (AIC) | 161.739 | 153.752 |
| Schwarz’s bayesian criterion (SBC) | 161.739 | 157.735 |
| Iterations | 1.000 | 3.000 |

| Test of the null hypothesis. |  |  |  |
| --- | --- | --- | --- |
| Statistic | DF | Chi-square | Pr > Chi² |
| -2 Log(Likelihood) | 4 | 15.98686129 | 0.003 |
| Score | 4 | 17.87847632 | 0.001 |
| Wald | 4 | 15.41367246 | 0.004 |
